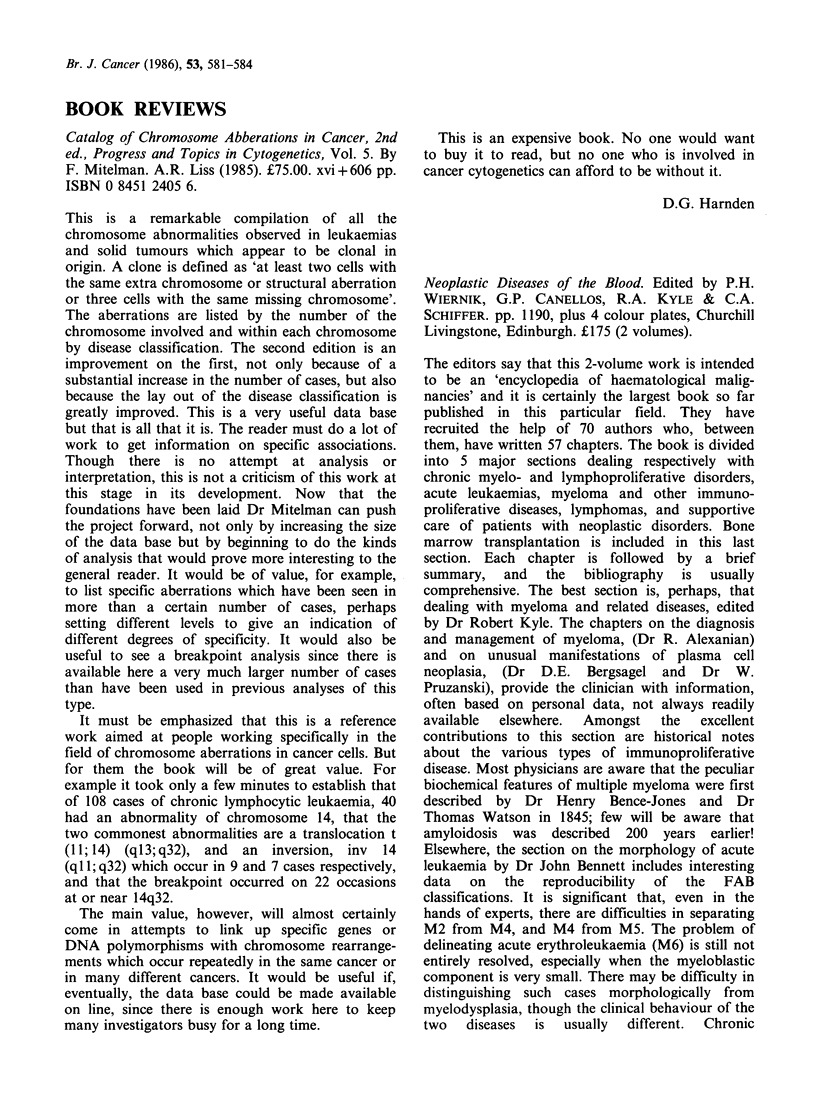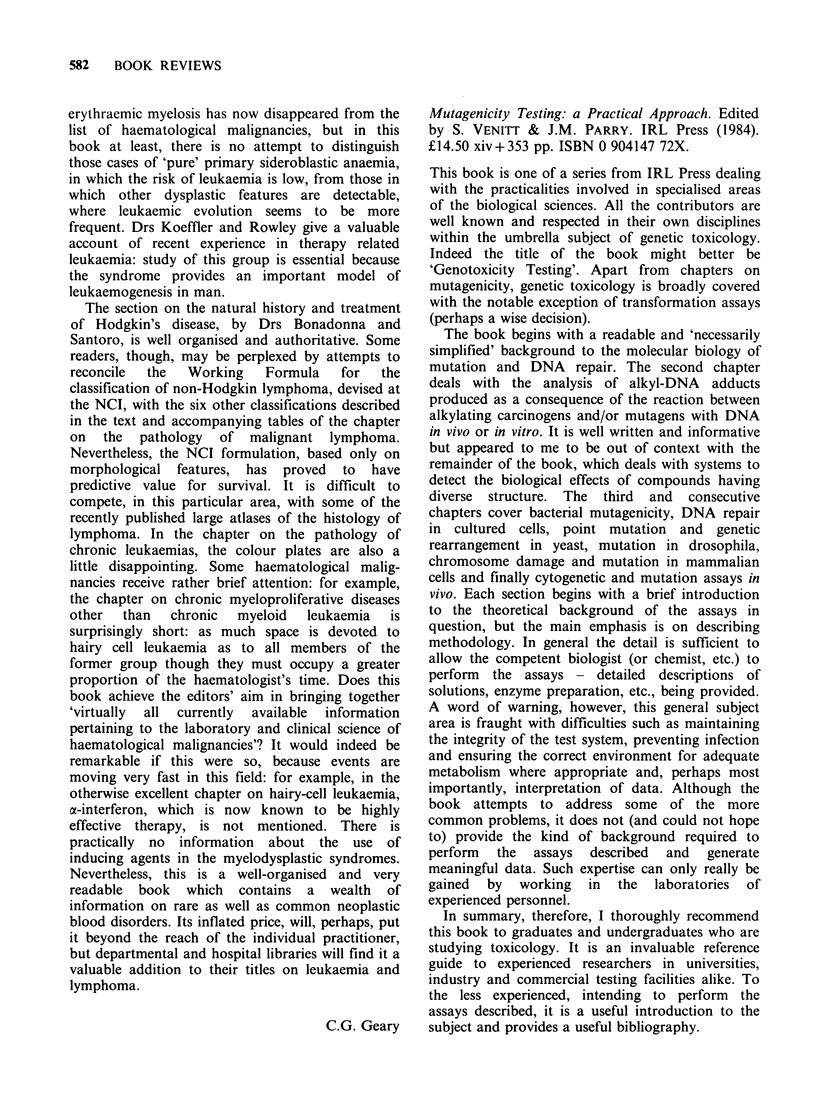# Neoplastic Diseases of the Blood

**Published:** 1986-04

**Authors:** C.G. Geary


*Neoplastic Diseases of the Blood.* Edited by P.H. Wiernik, G.P. Canellos, R.A. Kyle & C.A. Schiffer. pp. 1190, plus 4 colour plates, Churchill Livingstone, Edinburgh. £175 (2 volumes).

The editors say that this 2-volume work is intended to be an 'encyclopedia of haematological malignancies' and it is certainly the largest book so far published in this particular field. They have recruited the help of 70 authors who, between them, have written 57 chapters. The book is divided into 5 major sections dealing respectively with chronic myelo- and lymphoproliferative disorders, acute leukaemias, myeloma and other immunoproliferative diseases, lymphomas, and supportive care of patients with neoplastic disorders. Bone marrow transplantation is included in this last section. Each chapter is followed by a brief summary, and the bibliography is usually comprehensive. The best section is, perhaps, that dealing with myeloma and related diseases, edited by Dr Robert Kyle. The chapters on the diagnosis and management of myeloma, (Dr R. Alexanian) and on unusual manifestations of plasma cell neoplasia, (Dr D.E. Bergsagel and Dr W. Pruzanski), provide the clinician with information, often based on personal data, not always readily available elsewhere. Amongst the excellent contributions to this section are historical notes about the various types of immunoproliferative disease. Most physicians are aware that the peculiar biochemical features of multiple myeloma were first described by Dr Henry Bence-Jones and Dr Thomas Watson in 1845; few will be aware that amyloidosis was described 200 years earlier! Elsewhere, the section on the morphology of acute leukaemia by Dr John Bennett includes interesting data on the reproducibility of the FAB classifications. It is significant that, even in the hands of experts, there are difficulties in separating M2 from M4, and M4 from M5. The problem of delineating acute erythroleukaemia (M6) is still not entirely resolved, especially when the myeloblastic component is very small. There may be difficulty in distinguishing such cases morphologically from myelodysplasia, though the clinical behaviour of the two diseases is usually different. Chronic erythraemic myelosis has now disappeared from the list of haematological malignancies, but in this book at least, there is no attempt to distinguish those cases of 'pure' primary sideroblastic anaemia, in which the risk of leukaemia is low, from those in which other dysplastic features are detectable, where leukaemic evolution seems to be more frequent. Drs Koeffler and Rowley give a valuable account of recent experience in therapy related leukaemia: study of this group is essential because the syndrome provides an important model of leukaemogenesis in man.

The section on the natural history and treatment of Hodgkin's disease, by Drs Bonadonna and Santoro, is well organised and authoritative. Some readers, though, may be perplexed by attempts to reconcile the Working Formula for the classification of non-Hodgkin lymphoma, devised at the NCI, with the six other classifications described in the text and accompanying tables of the chapter on the pathology of malignant lymphoma. Nevertheless, the NCI formulation, based only on morphological features, has proved to have predictive value for survival. It is difficult to compete, in this particular area, with some of the recently published large atlases of the histology of lymphoma. In the chapter on the pathology of chronic leukaemias, the colour plates are also a little disappointing. Some haematological malignancies receive rather brief attention: for example, the chapter on chronic myeloproliferative diseases other than chronic myeloid leukaemia is surprisingly short: as much space is devoted to hairy cell leukaemia as to all members of the former group though they must occupy a greater proportion of the haematologist's time. Does this book achieve the editors' aim in bringing together 'virtually all currently available information pertaining to the laboratory and clinical science of haematological malignancies'? It would indeed be remarkable if this were so, because events are moving very fast in this field: for example, in the otherwise excellent chapter on hairy-cell leukaemia, α-interferon, which is now known to be highly effective therapy, is not mentioned. There is practically no information about the use of inducing agents in the myelodysplastic syndromes. Nevertheless, this is a well-organised and very readable book which contains a wealth of information on rare as well as common neoplastic blood disorders. Its inflated price, will, perhaps, put it beyond the reach of the individual practitioner, but departmental and hospital libraries will find it a valuable addition to their titles on leukaemia and lymphoma.

C.G. Geary